# Computational Fluid Dynamics Approach for Direct Nose-to-Brain Drug Delivery: A Systematic Review and Meta-Analysis

**DOI:** 10.3390/jpm15100447

**Published:** 2025-09-24

**Authors:** Priya Vishnumurthy, Thomas Radulesco, Gilles Bouchet, Alain Regard, Justin Michel

**Affiliations:** 1 Nemera La Verpillière, 69007 Lyon, France; alain.regard@nemera.net; 2 Aix Marseille University, CNRS, IUSTI, 13013 Marseille, France; 3 Aix Marseille University, APHM, CNRS, IUSTI, La Conception University Hospital, Oto-Rhino-Laryngology and Head and Neck Surgery Department, 13005 Marseille, France

**Keywords:** Nose-to-Brain, Computational Fluid Dynamics (CFD), olfactory region targeting, intranasal drug delivery, particle deposition

## Abstract

**Background/Objectives**: Optimizing drug deposition to the olfactory region is key in Nose-to-brain drug delivery strategies. However, findings from computational fluid dynamics (CFD) studies remain inconsistent concerning the parameters influencing olfactory deposition, limiting clinical translation and device optimization. This systematic review aims to identify robust CFD parameters for optimizing drug delivery to the olfactory region. **Methods**: A systematic review and meta-analysis were conducted following PRISMA guidelines, selecting studies reporting CFD simulations of nasal drug delivery with evaluation of olfactory deposition efficiency. The primary outcome was the correlation between each CFD parameter and olfactory deposition rate. Parameters included particle size, impaction parameter, flow rate, spray cone angle, insertion angle, injection velocity, head position, release position, and breathing pattern. Data were extracted and standardized, and statistical methods were used to assess correlations, heterogeneity, and potential biases in study results. **Results**: Smaller particle size (pooled r = −0.42) and lower impaction parameter (r = −0.39) were significantly associated with higher olfactory deposition. No consistent correlation was observed with breathing flow rate. Heterogeneity across studies was high (I^2^ > 90%). Funnel plots asymmetry suggested potential publication bias in particle-related outcomes. **Conclusions**: Particle characteristics, especially size and inertia, are the most critical determinants of olfactory deposition in CFD simulations. These findings support design optimization of nasal delivery devices targeting the olfactory region and underscore the need for standardized reporting and validation across CFD studies.

## 1. Introduction

Personalized medicine, tailoring drug delivery strategies to individual anatomical and physiological characteristics has become essential to improving therapeutic efficacy and minimizing systemic side effects. One such innovative approach is the direct Nose-to-Brain (NtB) pathway, which enables targeted transport of therapeutic agents to the central nervous system (CNS) via the olfactory region, and which is of interest for the treatment of neurodegenerative diseases.

Indeed, neurodegenerative diseases represent a growing global health burden [1], with limited treatment options due to the restrictive nature of the blood–brain barrier (BBB) [2]. The BBB, formed by tightly connected endothelial cells, pericytes and astrocytic end-feet ensures CNS homeostasis but severely restricts the entry of most therapeutic molecules. As a result, over 98% of small molecules and nearly all large biologics fail to cross this barrier in therapeutically relevant concentrations [3].

The direct Nose-to-Brain (NtB) pathway enables the direct transport of drugs to the CNS leveraging the olfactory nerves and thus has emerged as a promising alternative by bypassing the BBB and avoiding hepatic first-pass metabolism [4]. Drugs administered intranasally can reach the CNS through two main routes: an extracellular pathway, where molecules travel along perineural channels surrounding olfactory and trigeminal nerves, and an intracellular pathway, involving endocytosis and axonal transport within olfactory sensory neurons [5].This approach offers faster therapeutic onset, reduced systemic effects and improved patient compliance due to its non-invasive nature compared to conventional delivery methods [6].

Nevertheless, effective targeting of the olfactory region is a prerequisite for successful NtB drug delivery. Optimizing drug deposition in the region remains challenging due to the complex anatomy of the nasal cavity, variation in airflow dynamics and patient-specific factors [7]. While in vivo [8] and in vitro [9,10,11] approaches have contributed valuable insights, they are limited by ethical, technical and structural constraints particularly when evaluating drug transport to the upper nasal cavity [12].

Computational Fluid dynamics (CFD) has therefore emerged as an essential tool to simulate airflow and particle transport in the nasal cavity [13]. Yet, despite growing interest in CFD-based studies [14,15], findings remain inconsistent regarding which parameters most influence olfactory deposition. This variability hinders clinical translation and the development of optimized drug delivery devices.

A systematic meta-analysis represents a powerful approach to address these inconsistencies by aggregating findings from multiple CFD studies, reducing bias, and increasing statistical power to identify the parameters that most significantly affect drug deposition in the olfactory region. By clarifying these relationships, we contribute to the improvement of drug delivery strategies for Nose-to-Brain treatment in the context of personalized medicine for neurodegenerative disorders.

## 2. Materials and Methods

### 2.1. Study Selection

The PRISMA 2020 guidelines [16] were employed to conduct a systematic search, in January 2024, across ScienceDirect, PubMed and Web of Science using three distinct combinations of terms using Booleans operators: (1) (“Nose-to-Brain”) AND (“Computational Fluid Dynamics” OR “Computational”), (2) (“Nose-to-Brain” OR “Olfactory cleft” OR “olfactory region”) AND (“nasal rinse” OR “nasal irrigation” OR “nasal lavage” OR “nasal spray”) AND (“Computational Fluid Dynamics” OR “Computational”), and (3) (“Nose-to-Brain” OR “Olfactory cleft” OR “olfactory region”) AND (“drug delivery” OR “drug deposition”) AND (“Computational Fluid Dynamics” OR “Computational” OR “in silico”).

To determine the most appropriate keywords, a preliminary search was conducted in the selected databases to identify terms that were too broad and to refine those best suited to the aim of this study. No particular filter was applied. After duplicate removal, articles were screened by the corresponding author to select the studies to analyze. Inclusion criteria were studies using CFD simulations to assess olfactory drug deposition. Exclusion criteria included non-original articles, absence of CFD modelling, or lack of olfactory deposition data. The study has not been registered in any public registry source. No protocol has been prepared for this study.

### 2.2. Data Extraction

Selected articles were screened to identify the key parameters influencing the olfactory deposition. Articles not addressing the identified parameters were excluded from the study. When needed, data were standardized or recalculated using the following equations:

(1) Olfactory deposition = Particles deposited in olfactory region × 100/Total particle injected

and [17]

(2) Impaction parameter = Particle aerodynamic diameter^2^ × Breathing flow rate

One of the selected articles considered three different surfaces for the olfactory area [18]: small, medial, and large. Data for this particular article was treated as of three distinct studies, i.e., small (S), medial (M) and large (L).

The relationship between parameters and olfactory deposition reported in each studies were classified by the corresponding author as direct (an increase in one variable results in an increase in another variable), inverse (an increase in one variable results in another variable’s decrease and vice versa), nonlinear (an increase in one variable results in another variable’s increase or decrease until a certain point, then results in its decrease or increase), or null (there was no discernible relationship).

### 2.3. Statistical Analysis

For each selected study, statistical analyses were conducted on the extracted raw data. Analyses were not performed when the data were unavailable (e.g., provided only in graphical format) or insufficient (e.g., when fewer than five simulations were conducted). For a given parameter, the z-test is not conducted when fewer than five studies were available for comparison [19].

For key parameters with categorical subgroups, olfactory deposition was compared among subgroups with Kruskal–Wallis H test. For continuous key parameters, correlations with olfactory deposition were analyzed using Spearman’s correlation, which was interpreted according to the user’s guide to correlation coefficients [20] and pooled using a random-effect model.

Heterogeneity was assessed using Hunter–Schmidt τ^2^ [21], I^2^ [22], and Q statistics. When heterogeneity was detected, a prediction interval for the true outcomes was provided. Funnel plot asymmetry was evaluated using Egger’s regression [23] and Begg–Mazumdar tests [24]. Potential outlier and influential studies were identified using studentized residuals [25] and Cook’s distances [26], and sensitivity analyses were conducted if those latter were detected [27].

All statistical analyses were conducted using jamovi v2.3 [28], with significance set at *p* < 0.05.

## 3. Results

### 3.1. Study Selection

**Figure 1:**
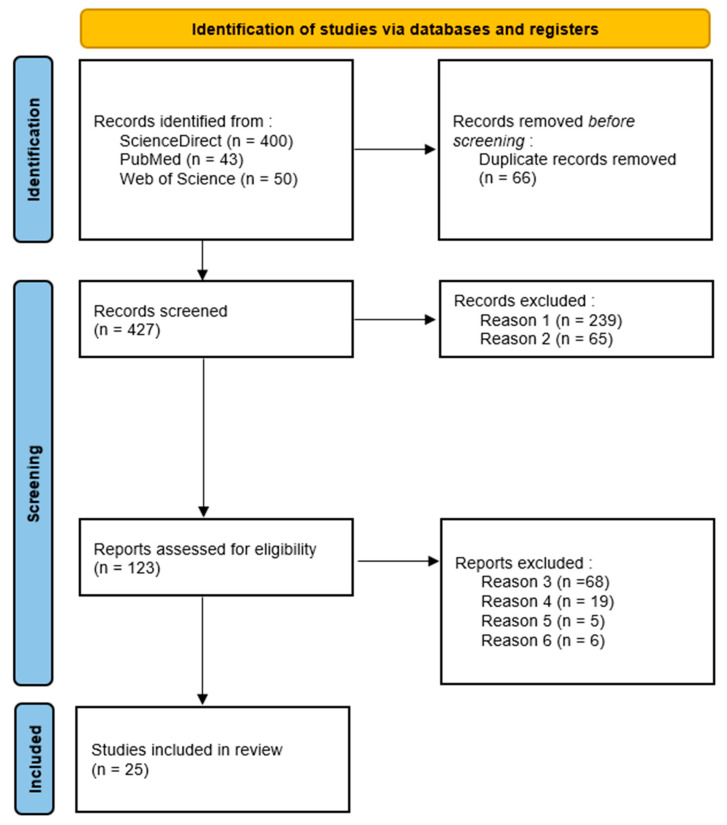
Study selection: PRISMA Flow diagram. Note: Reason 1: not full text research article; Reason 2: Out of scope according to the title and abstract of the paper; Reason 3: no use of CFD simulation; Reason 4: no consideration of the olfactory region; Reason 5: no consideration of at least one of the nine key parameters determined; Reason 6: no available data on olfactory deposition.

A total of 25 articles met the inclusion criteria. Nine key parameters were identified: three patient-related (breathing flow rate, breathing pattern, and head tilt position), three device-related (particle size, injection velocity, and spray cone angle) and three related to patient–device interaction (impaction parameter, release position, and sagittal insertion angle). The study selection process is outlined in Figure 1. Characteristics of the included studies are summarized in Table 1.

### 3.2. Descriptive Findings

The relationships between key parameters and olfactory deposition, as reported in each study, are compiled Table 2. There is no parameter for which articles are unanimous on the correlation with deposition in the olfactory zone. Reported deposition in the olfactory region varied widely (from 0% to 53%) (Table 1) and the best particle size identified for optimal delivery ranged from 0.001 to 60 µm (Table 2), reflecting high variability in model design and assumptions.

### 3.3. Meta-Analysis

Meta-analysis was conducted on the calculated Spearman’s correlation. All Spearman’s correlation coefficients calculated from the raw data were reported in Table 3.

#### 3.3.1. Patient-Dependent Parameters

• Breathing flow rate

**Figure 2:**
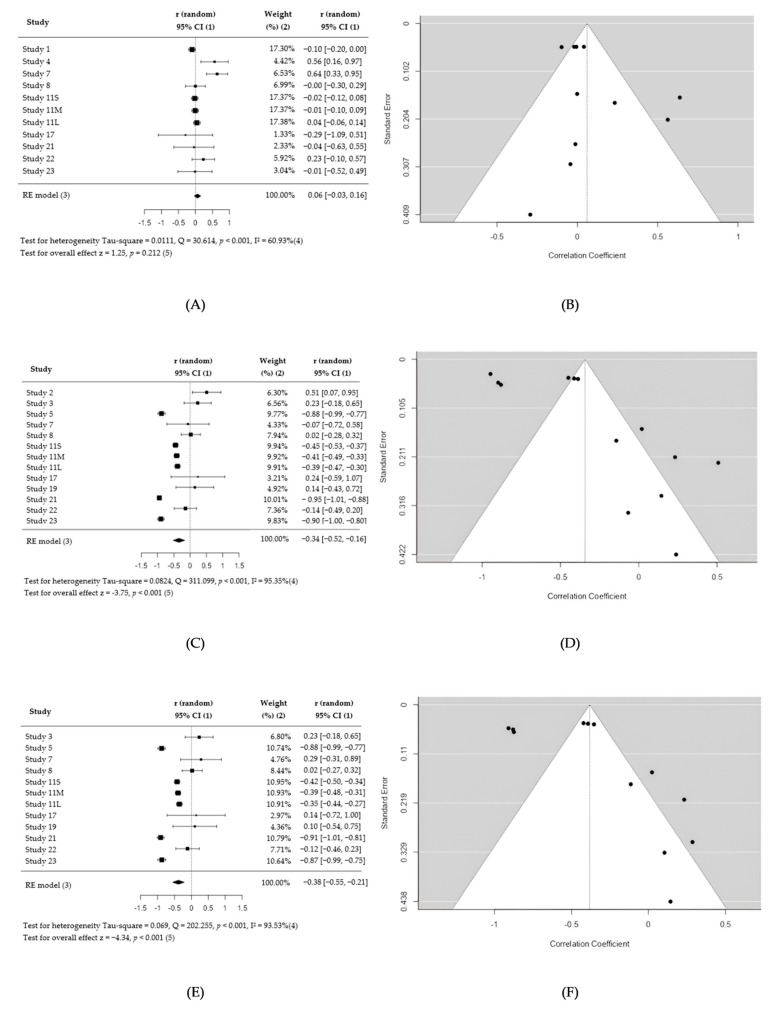
Forest plot and funnel plot of Flow rate (**A**,**B**), Particle size (**C**,**D**), and Impaction parameters (**E**,**F**). Note: References for all studies mentioned in the figure by their IDs can be found in Table 1. (1) Outcome of interest with a 95% confidence interval (CI) in picture and in number. (2) Influence of studies on overall effect, in percentage. (3) Overall random-effect (RE) model. On either side of the rhombus, the grey lines are the 95% prediction interval for the true outcome as tau^2^ > 0. (4) Heterogeneity. (5) The *p*-value of the test for overall effect.

Meta-analysis revealed no significant correlation between flow rate and olfactory deposition (r = 0.06, 95% CI −0.03 to 0.16) with moderate heterogeneity (I^2^ = 61%) (Figure 2A,B). Outlier analysis identified study 7 [34], but its exclusion did not alter conclusions on the true outcomes (Appendix A). No influential studies were identified based on Cook’s distances. Funnel plot asymmetry tests indicated no significant asymmetry (*p* = 1.0000 for the rank correlation and *p* = 0.0850 for the regression test).

• Breathing pattern

The Kruskal–Wallis H test, applied to data from study 19 [45], did not reveal statistically significant differences in olfactory deposition among inhalation, exhalation, and breath-holding conditions (*p* = 0.156).

• Head tilt position.

Analysis of data from study 4 [31] indicated no significant effect of head tilt on olfactory deposition (*p* = 0.183), confirming the study’s original findings.

#### 3.3.2. Device Dependent Parameters

• Monodispersed particle size

A strong negative correlation was observed between particle size and olfactory deposition (r = −0.34, 95% CI −0.52 to −0.16) with high heterogeneity (I^2^ = 95.35%). No outliers or influential studies were identified. The regression test revealed significant asymmetry (*p* < 0.0001) but not the rank correlation test (*p* = 0.6754). The funnel plot graphic revealed notable asymmetry (Figure 2C,D).

• Injection velocity and spray cone angle

Although a few studies reported significant correlations (Table 3), the limited number of available datasets prevented meta-analysis.

#### 3.3.3. Patient–Device Interaction Parameters

• Impaction parameter

A significant negative correlation was found between impaction parameters and olfactory deposition (r = −0.38, 95% CI −0.55 to −0.21) with high heterogeneity (I^2^ = 93.53%). No outliers or overly influential studies were identified. The regression test revealed significant asymmetry (*p* = 0.0003) but not the rank correlation test (*p* = 0.5452). The funnel plot graphic revealed notable asymmetry (Figure 2E,F).

• Release position

The Kruskal–Wallis H test, calculated from the raw data of study 4 [31], indicated no significant difference in olfactory deposition between different release positions (*p* = 0.442).

• Sagittal injection angle

Only one study reported a significant relationship between sagittal insertion angle and olfactory deposition (Table 3). Given the limited data and variability in methodology, no pooled analysis was performed.

## 4. Discussion

### 4.1. Summary of Key Findings

This meta-analysis reviewed 25 studies to assess which CFD parameters most significantly influence olfactory deposition. Three of the nine parameters, namely particle size, impaction parameter, and breathing flow rate, were included in the quantitative synthesis. Smaller particles and lower impaction parameters were significantly associated with higher olfactory deposition. In contrast, breathing flow rate alone showed no relationship with olfactory deposition. Other parameters, including injection velocity, spray cone angle, and sagittal insertion angle, could not be meta-analyzed due to limited or inconsistent data and should be interpreted cautiously.

### 4.2. Interpretation in Context of Literature

The observed negative correlation between particle size and olfactory deposition confirms prior hypotheses and simulation findings: low inertia particles better follow streamlines that reach the olfactory cleft [30]. Likewise, the impaction parameter, which reflects particle inertia [43], showed a negative association with olfactory deposition. High inertia particles are more likely to deviate from the airstream and impact proximal walls, depositing preferentially in the anterior part of the nasal cavity [18,43]. However, nuances exist. Some studies suggested that excessively small particles may follow airflow streamlines without depositing effectively in the olfactory region [18]. Conversely, smaller particles may offer faster dissolution and greater mucosal uptake potential, which can maximize the amount of drug delivered in the context of mucociliary clearance [14]. Conflicting results in the literature may stem from these competing aerodynamic and physiological process, as well as from modelling simplification of droplet agglomeration or particle size change during injection, factors often unaccounted for in CFD simulations [37].

Breathing flow rate, though frequently studied, did not show a significant correlation with olfactory deposition. This may be explained by the limited ventilation of the olfactory region regardless of global nasal flow [42]. Some studies have demonstrated that even high flow rates fail to meaningfully direct particles toward the upper nasal cavity unless impulse-driven or accompanied by a well-optimized injection trajectory [39,48,49]. This reinforces the idea that optimizing particle design may offer more leverage than adjusting inhalation conditions alone.

The impact of head position, release position, and breathing pattern remains inconclusive. While some studies suggested that inhalation or head tilt may improve deposition, our synthesis did not confirm a significant effect. This may be due to the strong influence of airflow streamlines over external positioning: for example, airflow reaching the olfactory zone often originates from the nose tip [52], which may explain why release near the tip enhances deposition regardless of nozzle orientation.

### 4.3. Source of Heterogeneity

High heterogeneity was observed across analyses, especially for particle size and impaction parameters. Several factors may have contributed to this variability. One is the wide range in how the olfactory surface was defined across studies, with surface areas ranging from 2.1% to 12.8% of the nasal cavity. Such disparities can affect both the calculated deposition percentages and the interpretation of a parameter’s relevance [18]. Additionally, differences in simulated patient anatomy, including age, surgical status, and inter-individual geometry, likely influenced deposition outcomes [17]. In some cases, the efficiency of olfactory deposition varied by two orders of magnitude across patient-specific models [39].

Another key source of heterogeneity stems from the diversity of CFD modeling approaches. Studies employed Lagrangian, Eulerian, and hybrid Euler–Lagrange methods, as well as volume of fluid (VOF) simulations for nasal irrigation. These differences in numerical resolution, boundary conditions, and spray modeling introduce additional variability that complicates meta-analysis. Furthermore, device types varied significantly, ranging from sprays to nebulizers, intubation, and exhalation-assisted devices, with differing spray dynamics and flow characteristics.

In line with such heterogeneity, prediction intervals were reported to emphasize the dispersion of true effects cross settings and to temper over-interpretation of pooled means.

Subgroup analysis could help explore this further but was not feasible here due to the limited number of studies per subgroup.

### 4.4. Potential Publication Bias

While formal statistical tests were not always concordant, visual funnel plot asymmetry was evident for particle size and impaction parameter, suggesting potential publication bias. This asymmetry implies that the pooled effect for these parameters may be overestimated and that studies with nonsignificant findings may be underrepresented. Limited access to raw numerical data (graph-only reporting) reduced the number of studies entering some analyses that may have amplified small-study effects.

Because each synthesis included few studies and exhibited substantial heterogeneity, no correction methods, such as the trim-and-fill method, were applied, given that those methods are unreliable under these conditions. However, it should be noted that the true effect of particle size and impaction parameter may be smaller than the pooled estimates.

### 4.5. Limitations

This study focused on nine individual parameters, but many CFD variables are interdependent. For example, spray cone angle and particle size may interact depending on flow rate, as observed in some studies [29,39,41]. Additionally, no study integrated all nine parameters simultaneously.

Clinically, our findings support a focus on particle engineering for enhancing olfactory drug delivery. Designing monodisperse particles with low inertia may maximize olfactory deposition regardless of external factors like flow rate or nozzle position. However, device design must also account for patient comfort, mucociliary clearance, and overall drug pharmacokinetics, which were not addressed in this analysis.

Future research using multivariate CFD simulations, or machine learning models trained on harmonized large datasets, could help identify optimal parameter combinations for targeted delivery. In vivo validation studies and standardized CFD reporting will also be essential to translate these insights into patient-specific NtB therapies.

## 5. Conclusions

This meta-analysis identified particle size and impaction parameter as the most significant CFD predictors of olfactory drug deposition, while breathing flow rate showed no consistent effect. However, high heterogeneity, methodological variability, and potential publication bias across studies indicate that the true effects may be smaller than pooled estimates and highlight the need for standardized CFD protocols, multivariate analysis approaches, and experimental validation. Future research should focus on identifying optimal parameter combinations and translating silico predictions into clinically meaningful delivery strategies tailored to individual patient anatomies.

## Figures and Tables

**Table 1:** Characteristics of the included studies and parameters investigated in each study. Note: NA: the data were not available; EDM: Exhalation-assisted aerosol Delivery Method; VOF: Volume of Fluid; CT: Computed Tomography; MRI: Magnetic resonance imaging; A: Respiratory flow rate; B: Breathing pattern; C: Head tilt position; D: Monodispersed particle size; E: Injection velocity; F: Spray cone angle; G: Release position; H: Sagittal injection angle; I: Impaction parameter; x: the study has investigated the parameter; +: the study did not investigated the parameter but the correspondent author calculated the data with Equation (2); *: data were sufficient and available to conduct statistical analysis.

Study ID	Source Selected	Sample Size	3D Model	Mean Age of the Sample	Male Ratio of the Sample	Olfactory Surface (% of the Total Nasal Cavity)	Numerical Model Selected	Device Simulated	A	B	C	D	E	F	G	H	I	Maximum Olfactory Deposition (%)	Reference
1	CT	1	Realistic	48	1	NA	Lagrangian	Nasal spray	x *				x *	x *		x *		1.60	[29]
2	CT	1	Realistic	48	1	NA	Lagrangian	Inhaled particles				x *						2.5	[30]
3	1 MRI + 2 CT	3	2 realistic and 1 standardized	44.43	1	NA	Lagrangian	Inhaled particles				x *					x *	1.33	[17]
4	CT	6	Realistic and operated	NA	NA	NA	Euler– Lagrange	Nasal spray	x *		x *		x *		x *			36.33	[31]
5	CT	3	Realistic	62	1/3	3–5.9	Lagrangian	Inhaled particles				x *					+ *	1.4	[32]
6	CT	1	Realistic	54	0	NA	Lagrangian	Inhaled particles	x *			x			x			3.1	[33]
7	CT	1	Standardized	45.3	13/30	8	Euler– Lagrange	Vibrating mesh nebulizer	x *			x *	x *				+ *	8.5	[34]
8	CT	1	Realistic	80	NA	NA	Lagrangian	Inhaled particles	x *			x *					+ *	18.45	[35]
9	CT	1	Realistic	59	0	NA	Lagrangian	EDM	x	x		x						0	[36]
10	CT	1	Realistic	6	0	3.8	Euler– Lagrange	Nasal spray					x	x				<6	[37]
11 S	CT	30	Realistic	46.5	1/2	2.1–3.2	Lagrangian	Inhaled particles	x *			x *					x *	1.39	[18]
11 M	CT	30	Realistic	46.5	1/2	4.3–6.4	Lagrangian	Inhaled particles	x *			x *					x *	5.94	[18]
11 L	CT	30	Realistic	46.5	1/2	8.6–12.8	Lagrangian	Inhaled particles	x *			x *					x *	6.28	[18]
12	CT	12	Realistic	5	1/2	NA	Lagrangian	Inhaled particles	x									0.46	[38]
13	CT	7	Realistic	60	5/7	2.8	Lagrangian	Nasal spray				x	x		x			<25	[39]
14	MRI	1	Realistic	53	1	NA	Euler– Lagrange	Nasal spray				x	x		x	x		<4	[40]
15	CT	1	Realistic	NA	NA	NA	Discrete phase model	Nasal spray	x			x	x	x	x			<1	[41]
16	MRI	3	Realistic and modification	28	1	NA	Discrete phase model	Inhaled particles	x									<5	[42]
17	MRI	1	Realistic	53	1	NA	Euler– Lagrange	Inhaled particles	x *		x	x *					x *	30.8	[43]
18	CT	1	Realistic	25	0	NA	VOF	Squeeze-bottle nasal irrigation			x							-	[44]
19	MRI	1	Realistic	53	1	8	Lagrangian	Deep or vestibular intubation		x *		x *			x		+ *	1.09	[45]
20	MRI	1	Realistic	53	1	8	Lagrangian	Nasal spray			x	x		x	x	x		<6	[46]
21	CT	1	Realistic	48	1	10.5	Lagrangian	Inhaled particles	x *			x *					+ *	3.02	[47]
22	MRI	1	Realistic	53	1	NA	Lagrangian	Inhaled particles	x *			x *					+ *	3.97	[48]
23	MRI	1	Realistic	53	1	NA	Lagrangian	Inhaled particles	x *			x *			x		+ *	53.21	[49]
24	MRI	1	Realistic	53	1	8	Lagrangian	Inhaled particles				x				x		<2	[50]
25	CT	8	Realistic and virtual surgery	4	3/4	10	Lagrangian	Inhaled particles	x	x		x						2.78	[51]

**Table 2:** Relationship between key parameters and olfactory deposition. Note: Studies that did not consider continuous parameters were not included in the Table. NC means that the study found no correlation between olfactory deposition and particle size, conducting to no estimation of the best particle size. MSR: Targeted injection from the Medial Superior Region of the nostril enhances olfactory deposition; IR: Targeted injection from the Inferior Region of the nostril enhances olfactory deposition.

Study ID	Flow Rate	Breathing Pattern	Head Position	Particle Size	Best Particle Size (µm)	Injection Velocity	Spray Cone Angle	Impaction Parameter	Sagittal Insertion Angle	Release Position	Reference
1	Null					Null	Null		Null		[29]
2				Non-linear	10						[30]
3				Inconclusive	NC			Direct			[17]
4	Direct		Null			Inverse				Null	[31]
5				Inverse	<0.007						[32]
6	Non-linear			Non-linear	0.001					MSR	[33]
7	Direct			Null	Between 0.001 and 0.007	Direct					[34]
8	Direct			Non-linear	<0.02 and >10						[35]
9	Inverse	Inconclusive		Inconclusive	NC						[36]
10						Null	Inverse				[37]
11	Direct			Non-linear	20			Inverse			[18]
12	Inverse										[38]
13				Non-linear	Between 20 and 30	Inverse				MSR	[39]
14				Null	NC	Null			Null	MSR	[40]
15	Non-linear			Non-linear	25	Inverse	Direct or inverse depending on the airflow rate			MSR	[41]
16	Direct										[42]
17	Non-linear			Direct	10			Non-linear			[43]
18			45° backward head tilt position enhance olfactory deposition								[44]
19		Higher olfactory deposition rate during inhalation. Lowest deposition rate during breath holding.		Inverse	< 30					MSR	[45]
20			Vertex-to-floor position is supposed to increase olfactory deposition	Non-linear	60		Inverse		Direct	IR	[46]
21	Direct			Non-linear	0.002						[47]
22	Non-linear			Non-linear	Between 0.01 and 0.1 and between 10 and 20						[48]
23	Direct			Inverse	0.001					MSR	[49]
24				Inconclusive	NC				Inverse		[50]
25	Inverse	Higher deposition rate during inhalation and lower deposition rate during exhalation		Inverse	0.001						[51]

**Table 3:** Spearman’s correlation between continuous parameters and olfactory deposition. Note: In bold, significant correlations. * Significant. ** strong significance. *** very strong significance. NA means that not enough data was available to conduct Spearman’s correlation analysis. In grey, the studies that did not consider a specific parameter. In green, the studies where Spearman’s correlation corroborate the relationship found by the authors. In yellow, the studies where Spearman’s correlation was not in line with the relationship found by the authors.

Study ID	Flow Rate			Monodispersed Particle Size			Injection Velocity			Spray Cone Angle			Sagittal Injection Angle			Impaction Parameter			Reference
	Event	r	*p*	Event	r	*p*	Event	r	*p*	Event	r	*p*	Event	r	*p*	Event	r	*p*	
1	384	−0.098	0.056				384	−0.003	0.961	384	0.202 ***	<0.001	384	−0.1 **	0.008				[29]
2				12	0.508	0.092													[30]
3				21	0.232	0.312										21	0.232	0.312	[17]
4	12	0.563	0.056				12	−0.631 *	0.028										[31]
5				18	−0.879 ***	<0.001										18	−0.879 ***	0.001	[32]
6	3	0.866	0.333	NA	NA	NA													[33]
7	15	**0.638** *	0.011	10	−0.067	0.865	15	0.638 *	0.011							10	0.286	0.301	[34]
8	45	−0.001	0.994	45	0.02	0.897										45	0.023	0.883	[35]
9	NA	NA	NA	NA	NA	NA													[36]
10							NA	NA	NA	NA	NA	NA							[37]
11	400	−0.02	0.696	400	−0.449 ***	<0.001										400	−0.422 ***	0.001	[18]
11	400	−0.006	0.912	400	−0.412 ***	<0.001										400	−0.393 ***	0.001	[18]
11	400	0.041	0.409	400	−0.387 ***	<0.001										400	−0.354 ***	0.001	[18]
12	NA	NA	NA																[38]
13				NA	NA	NA	NA	NA	NA										[39]
14				NA	NA	NA	NA	NA	NA				NA	NA	NA				[40]
15	NA	NA	NA	NA	NA	NA	NA	NA	NA	NA	NA	NA							[41]
16	NA	NA	NA																[42]
17	6	−0.293	0.573	6	0.239	0.648										6	0.143	0.803	[43]
18																			[44]
19				12	0.145	0.653										10	0.104	0.774	[45]
20				NA	NA	NA				NA	NA	NA	NA	NA	NA				[46]
21	12	−0.043	0.894	12	−0.946 ***	<0.001										12	−0.909 ***	0.001	[47]
22	32	0.233	0.199	32	−0.143	0.434										32	−0.115	0.53	[48]
23	16	−0.012	0.964	16	−0.897 ***	<0.001										16	−0.874 ***	0.001	[49]
24				NA	NA	NA							NA	NA	NA				[50]
25	NA	NA	NA	NA	NA	NA													[51]

## Data Availability

All the raw data used in this article have been extracted from other studies. Please refer to the corresponding authors of those studies to get the raw data. All the statistical data are provided in the Tables and the Figures of this study.

## Appendix A

**Table A1:** Sensitivity analysis of the meta-analysis between flow rate and olfactory deposition for the detected potential outlier, study 7.

Data	With Outlier Study	Without Outlier Study
Random-Effect Model		
Estimate	0.0607	0.00145
SE	0.0487	0.0331
Z	1.25	0.0440
*p*	0.212	0.965
CI Lower Bound	−0.035	−0.063
CI Upper Bound	0.156	0.066
Heterogeneity Statistics		
Tau	0.105	0.050
Tau^2^	0.0111 (SE = 0.0081)	0.0025 (SE = 0.0036)
I^2^	60.93%	27.69%
Q	30.614	14.386
*p*	<0.001	0.109
Publication Bias assessment		
Begg and Mazumdar Rank Correlation	*p* = 1.000	*p* = 0.727
Egger’s Regression	*p* = 0.085	*p* = 0.227
